# Gene Expression in the Three-Spined Stickleback (Gasterosteus aculeatus) of Marine and Freshwater Ecotypes

**Published:** 2018

**Authors:** S. M. Rastorguev, A. V. Nedoluzhko, N. M. Gruzdeva, E. S. Boulygina, S. V. Tsygankova, D. Y. Oshchepkov, A. M. Mazur, E. B. Prokhortchouk, K. G. Skryabin

**Affiliations:** National Research Center “Kurchatov Institute”, Kurchatov Sq. 1, Moscow, 123182, Russia; Institute of Bioengineering, Research Center of Biotechnology of the Russian Academy of Sciences, Leninsky Ave. 33, bldg. 2, Moscow, 119071, Russia; Faculty of Biology, Lomonosov Moscow State University, Leninskie Gory 1–12, Moscow, 119991, Russia; Institute of Cytology and Genetics of the Siberian Division of Russian Academy of Sciences, Lavrentieva Ave. 10, Novosibirsk, 630090, Russia

**Keywords:** hree-spined stickleback, Gasterosteus aculeatus, gene expression, differential expression, RNA-seq, osmoregulation, GO-analysis, speciation

## Abstract

Three-spine stickleback *(Gasterosteus aculeatus) *is a well-known model organism that is routinely used to explore microevolution processes and speciation, and the number of studies related to this fish has been growing recently. The main reason for the increased interest is the processes of freshwater adaptation taking place in natural populations of this species. Freshwater three-spined stickleback populations form when marine water three-spined sticklebacks fish start spending their entire lifecycle in freshwater lakes and streams. To boot, these freshwater populations acquire novel biological traits during their adaptation to a freshwater environment. The processes taking place in these populations are of great interest to evolutionary biologists. Here, we present differential gene expression profiling in *G. aculeatus *gills, which was performed in marine and freshwater populations of sticklebacks. In total, 2,982 differentially expressed genes between marine and freshwater populations were discovered. We assumed that differentially expressed genes were distributed not randomly along stickleback chromosomes and that they are regularly observed in the “divergence islands” that are responsible for stickleback freshwater adaptation.

## INTRODUCTION

**Fig. 1:**
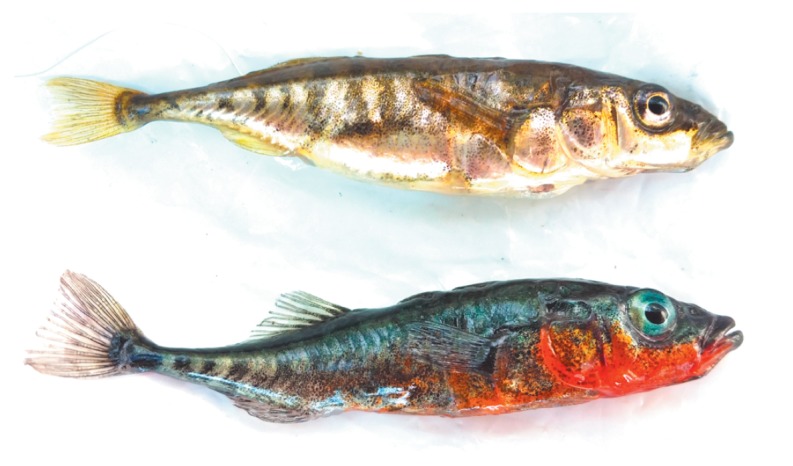
Three-spined stickleback. Freshwater form. Stickleback female (top), stickleback male in breeding dress (bottom)

Three-spined stickleback *(Gasterosteus aculeatus)* (*Fig. 1*) is a well-known model organism that is commonly used to explore the adaptive speciation process [1], since its marine form colonizes freshwater areas across the entire coastline of the Northern Hemisphere. A marine population of three-spined stickleback usually uses freshwater streams and lakes for spawning. However, isolation in a new habitat leads to the formation of a freshwater resident population, whose morphology changes over time as other features that make survival possible in new conditions develop. This feature makes it possible to use this small fish as a model for studying adaptive evolution in similar habitat conditions.

To date, a number of investigations have been published on the genome-wide changes that occur in three-spined stickleback during the process of adaptive speciation [2-4], which describe genomic “divergence islands” where the nucleotide substitutions characteristic of the freshwater form are concentrated. There are studies that describe the differential expression of stickleback genes in the kidneys of marine and freshwater specimen and the changes that occur after the introduction a freshwater specimen to a marine environment [5], as well as changes in the muscles, epithelial and bone tissues of marine and freshwater stickleback populations in studies of the plasticity of gene expression during colonization of freshwater habitats [6]. In addition, the differential expression of *G. aculeatus* genes in the tissues of the kidney and spleen in lake and river fish was evaluated in a study of the immune response to parasitic fauna [7].

The differences in the expression of genes in marine and freshwater forms have been widely studied in other models. Diadromous fish are especially suitable for this type of research, since they can live in both marine and fresh water and have physiological mechanisms for adaptation to water of differing salinity. In addition, global changes in gene expression in the marine and freshwater forms of such species as *Plecoglossus altivelis *ayu [8], Japanese river acne *Anguilla japonica* [9], European acne *A. anguilla *[10], tilapia *Oreochromis mossambicus *[11, 12], *Fundulus heteroclotus *[13], common laurel *Dicentrarchus labrax *[14], sockeye *Oncorhynchus nerka *[15], arctic char *Salvelinus alpinus* [16] are studied by the RNA-seq method. In most cases, the RNA-seq method is used to evaluate subsequent changes in gene expression after changes in the external environment with gills as the target tissue. The categories of gene ontology (GO, Gene Ontology), which are enriched in experimental groups, have been identified, and metabolic and biochemical pathways, which play an important role in adaptation to changes in osmotic conditions, have been proposed. In particular, it has been shown that changes in osmotic conditions lead to changes in the expression of the genes that encode transport proteins and ion channels [12], the genes responsible for cell growth and proliferation, apoptosis and molecular transport, protein synthesis, and energy metabolism [9, 11, 13]. The active involvement of transcription factors in this process [15], which indicates an extensive effect of changing osmotic conditions on gene expression, deserves special mention.

Examination of gene expression can shed light on such fundamental problems of genetics as the connection between structure and functions in the eukaryotic genome. It is generally believed that genes in the eukaryotic genome are distributed randomly without forming any functional clusters similar to bacterial operons. However, there is evidence that this statement is false: statistical analysis of genome-wide data and transcription analysis data have demonstrated that genes in the eukaryotes genome are not distributed randomly but are organized into co-expressed clusters [17, 18]. Moreover, it has been shown that the genes of Arctic char [16], orthologous to the genes of threespined stickleback, which are differentially expressed in the gills of fish from fresh and marine water, are much closer to each other than they would have been in a random arrangement, which confirms the hypothesis of a cluster organization of the eukaryotic genome. However, it would be of interest to compare these data with gene expression in three-spined stickleback.

In this paper, we present the results of a RNA-seq analysis of the genes expressed in the gills of marine and freshwater forms of three-spined stickleback; we have identified genes whose expression levels differ significantly in these two forms. We used gills as the target tissue, because they play an important role in the osmotic balance, and they are easy to isolate, which reduces the errors associated with the collection of material for the study. We elucidated the genomic localization of differentially expressed genes. For each chromosome, we calculated the ratio of the intergenic distances of such genes to the mean in the chromosome. We additionally performed functional and Gene Ontology analyses, identified the biochemical pathways enriched with the identified genes, and compared the data obtained with previously published data for other species. The ratio of differentially expressed genes to the genomic “divergence islands” involved in the adaptation of three-spined stickleback to a freshwater habitat was determined [4].

## EXPERIMENTAL

Samples of marine three-spined stickleback (hereinafter referred to as “M”) were collected from the White Sea, near the N.A. Pertsov White Sea Biological Station of Moscow State University (WSBS, MSU, Primorskiy settlements, Murmansk Region). Freshwater samples (hereinafter referred to as “F”) were collected from the Machinnoye Lake, not far from the village of Tchkalovsky, Loukhskiy district, Republic of Karelia. Based on its location above sea level, the approximate age of the lake after desalination is 700 years [4, 19]. The lake contains only resident freshwater forms, since the stream leading from the lake into the sea is swampy and impassable for anadromous stickleback. In addition, the risk of collection error was reduced due to the significant morphological differences between the marine and freshwater forms [20]. To synchronize the physiological status of the samples, only males in breeding dress were selected.

The collected samples were kept for 4 days, each in their own water in the aquariums at the WSBS to synchronize the stress factor, which may differ depending on the collection conditions. Afterwards, the gills were isolated and fixed in a IntactRNA® reagent (“Eurogen”, Russia).

RNA from the gill tissue of *G. aculeatus *(five samples from each experimental group) was isolated according to a standard protocol using a TRIzol® reagent (Invitrogen, USA). The RNA concentration for each sample was determined using a BioAnalyzer 2100 (RNA 6000 Nano Kit) (Agilent, USA).

To obtain cDNA libraries, cDNA was first synthesized on the RNA template using a set of Mint® reagents (“Eurogen”, Russia) according to the manufacturer’s instructions. Then, 10 indexed pair-end libraries for sequencing on Illumina analyzers (Illumina, USA) were prepared using the NEBNext Library Prep Kit for Illumina (NEB, UK). The concentration and purity of the libraries were determined using an Agilent Bioanalyzer 2100 instrument (Agilent Technologies, USA), followed by sequencing on Illumina HiSeq 1500 with a length of 2 × 75 nucleotides.

To identify the genes which are differentially expressed in the marine and freshwater samples of threespined stickleback, the Illumina nucleotide reads were mapped on the G. aculeatus reference genome from the Ensembl database (BROAD S1, Feb 2006, assembly 81; http://www.ensembl.org) [21] using the bowtie2 software package [22] with the set of parameters “-very-sensitive-local.” As a result, SAM (Sequence Alignment/Map) files [23] were produced, which were further processed (compression, sorting, indexing) using the SAMtools package [23, 24]. The relative activity of each gene was determined according to the coverage of this gene by nucleotide reads on the reference genome after the mapping of each library. The coverage was determined using the coverageBed tool from the bedtools software package [25], using the bed-file with gene coordinates from Ensembl, and an indexed bam file obtained as a result of mapping of the nucleotide reads. The mapping data for each library was collated in a single table using a perl script. Statistical analysis of differential expression was performed using the edgeR package [26] of the R software for statistical computations (http://www.r-progect.org).

**Table 1:** Number of Illumina reads

Library	Number of clusters	Number of reads	Reads mapped on genes	Total for marine and freshwater forms	
				produced	mapped
M2	10566712	21133424	17993109	85438630	74093974
M3	10577457	21154914	18161521		
M4	10262893	20525786	18489001		
M5	11312253	22624506	19450343		
F1	13523593	27047186	24692145	110690898	103570453
F2	15715663	31431326	28960967		
F4	13359490	26718980	26307475		
F5	12746703	25493406	23609866		

^*^Sequencing statistics. The number of Illumina reads were obtained for each RNA-library and for the marine and freshwater stickleback populations altogether.

The analysis of gene ontologies (GO-Gene Ontology) and the analysis of biochemical pathways were carried out using the PANTHER (Protein Annotation through Evolutionary Rela- tionships) software (http://pantherdb.org) [27], after translating the Ensembl ID of stickleback genes into human orthologic genes with the help of BioMart Ensembl service, because this software does not use the genome of three-spined stickleback as a reference for searching for enriched GO categories. This utility uses the GO PANTHER library, based on models that use the hidden Markov chain algorithm to identify enrichment categories. Both “full” and reduced GO slim categories are used.

**Fig. 2:**
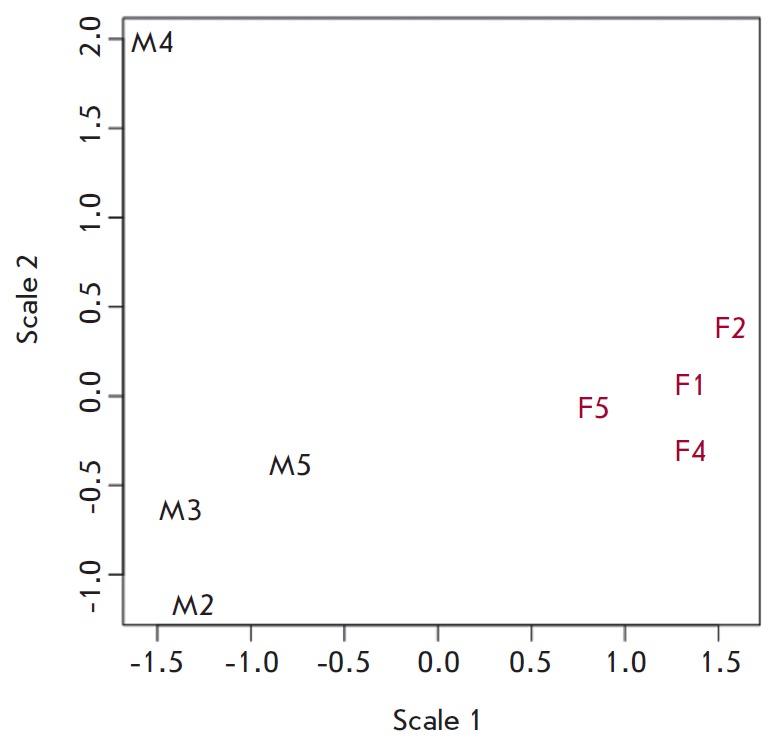
Multi-dimensional scaling (MDS) plot of marine and freshwater stickleback based on normalized gene expression profiles in RNA-seq libraries. Marine samples are marked with red dots (M), freshwater samples are marked with blue ones. The indexes correspond to the RNA-libraries index numbers

The intergenic distances for the complete set of genes of three-spined stickleback were compared with the distances between the genes responsible for osmoregulation using a perl script. Using the coordinates of the genes on each chromosome (indicated in bed-files from Ensembl’s ftp server), the distance from each gene to all other genes of a given chromosome was measured and the same was done for all genes of the genome, resulting in an array of intergenic distances in nucleotides. A similar procedure was carried out for those genes that were differentially expressed in the gills of the marine and freshwater forms. We transferred the two acquired arrays to the t.test function of the R software for statistical computation, producing the difference indices for the two arrays.

**Fig. 3:**
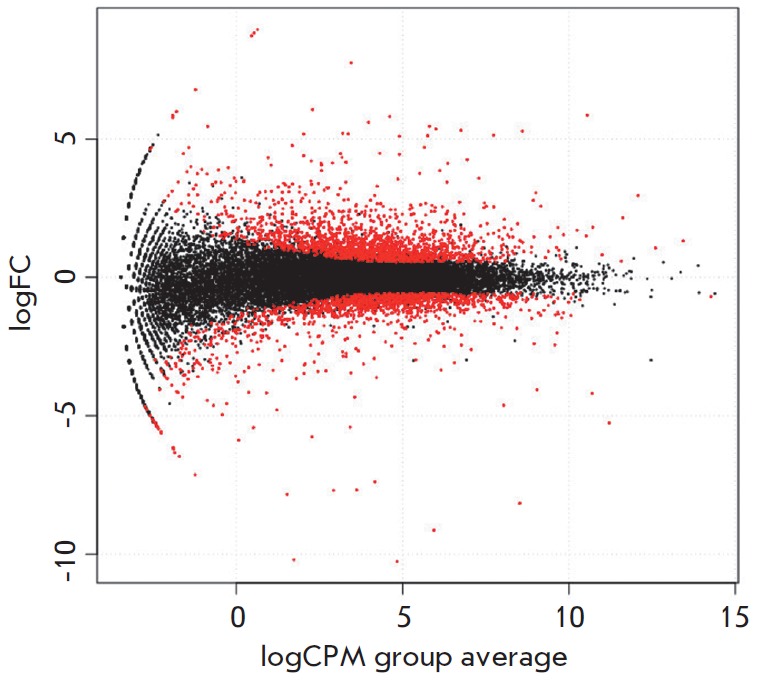
Genes differentially expressed in three-spined stickleback gills. The dependency of logCPM (logarithm of count per million) on logFC (logarithm of fold change). These are binary logarithms. Marine vs freshwater differential genes, with more than 95% statistical support, marked with red dots

The work was carried out using the equipment of the Center for Collective Use “Complex for Modeling and Data Processing of Mega-Class Research Facilities” of the Kurchatov Institute, http://ckp.nrcki.ru

**Table 2:** Genes with the widest difference in expression level between marine and freshwater stickleback samples

Ensembl gene ID	logFC	logCPM	P-value	FDR
ENSGACG00000013714	-4.193912	10.693346	2.116876e-51	4.753656e-47
ENSGACG00000011986	-5.259545	11.215562	6.575861e-51	7.383376e-47
ENSGACG00000001275	3.860307	6.117474	2.371864e-46	1.775419e-42
ENSGACG00000014967	4.253744	6.943885	6.017277e-41	3.378099e-37
ENSGACG00000018764	-4.056880	9.038716	2.477170e-40	1.112547e-36
ENSGACG00000014959	4.706814	5.650018	7.523387e-40	2.815753e-36
ENSGACG00000003404	-4.617256	8.036567	5.344099e-37	1.714387e-33
ENSGACG00000001373	3.762800	5.512202	4.071101e-35	1.061745e-31
ENSGACG00000019813	5.816259	4.613614	4.255301e-35	1.061745e-31
ENSGACG00000014691	4.449242	4.901331	9.436192e-35	2.118991e-31

Note. logFC – binary logarithm of expression fold change, logCPM count per million – expression level characteristic, P-value – difference in expression, FDR – (false discovery rate) – P-value, normalized for multiple comparisons.

## RESULTS AND DISCUSSION

tially, five samples of stickleback from marine and freshwater populations were selected for the study of differential expression. However, the preparation of cDNA libraries revealed that two samples (one from each population) were of poor quality and they were excluded from the subsequent analysis. Therefore, four cDNA libraries, suitable for sequencing on the Illumina platform, were obtained for each group

The total number of reads of 75 nucleotides in length was 85438630 and 110690898 in the libraries from the marine and freshwater samples, respectively. Nucleotide reads (177664427 in total) were mapped on the genes annotated in the G. *aculeatus* genome from the Ensembl database. The information on the number of nucleotide reads obtained as a result of the experiment and the mapping statistics are presented in *Table 1*.

After mapping of the data on the G. aculeatus genome, the nucleotide reads mapped on each of the annotated three-spined stickleback genes were counted and the activity of each gene was normalized using the edgeR package.

A MDS (Multi Dimensional Scaling) graph was constructed using the data on the coverage of annotated genes; in this graph, the arrangement of the samples corresponds to the differences in the expression of their genes. There were significant differences in the expression of genes in marine and freshwater stickleback samples. At the same time, samples of each group formed a fairly tight cluster (with the exception of the M4 marine sample), which indicates good synchronization of the physiological processes between the samples studied (*Fig. 2*).

**Fig. 4:**
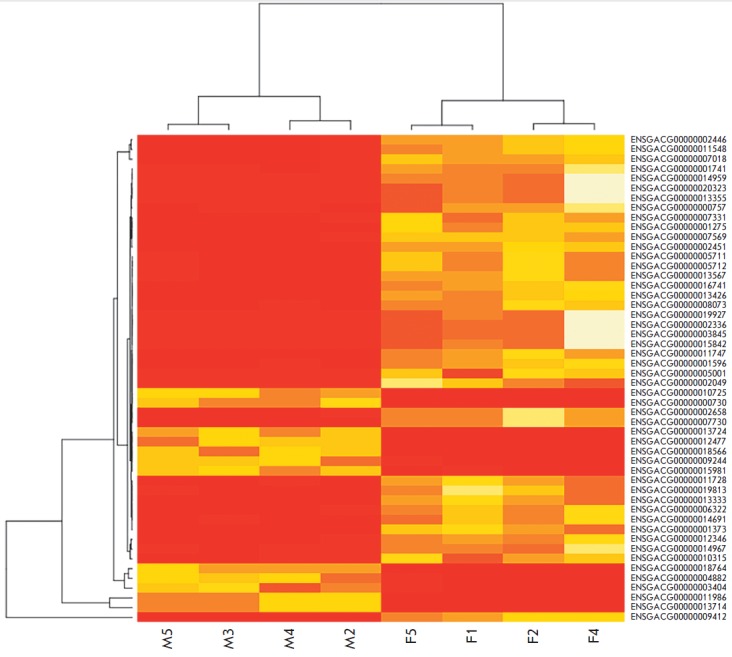
Heat map diagram of the 50 most differentially expressed genes in the gill samples of marine and freshwater three-spined sticklebacks. The expression values are normalized using CPM (count per million) measure. The heat map indicates up-regulation (red) and down-regulation (yellow). The columns represent individual tissue samples (M – marine, F – freshwater), and rows represent gene names. The marine and freshwater stickleback samples are grouped separately Differences in the grouping of marine and freshwater specimen are visible

Differential expression was established using the edgeR package [26], which calculates the variance of the expression index for each gene. Genes were considered differently expressed if the difference between their activity and the mean was significantly higher than the variance. When calculating differential expression, the degree of gene activity is also important: for poorly expressed genes, the deviation from the mean should be higher for the difference in expression to be recognized as reliable. *Figure 3* illustrates the information presented above: differentially expressed genes (red dots) are genes whose expression not only deviated significantly from the mean, but also was on a fairly high level.

**Table 3:** The intergenic distances for a whole gene set and differentially expressed genes in three-spined stickleback.

Chromosome	Length, b.p. according to Ensembl	Number of genes	Number of differentially expressed genes	Mean intergene distance, b.p.	Mean between differentially expressed genes, b.p.	Р-value^**^
group I	28185914	1647	150	9760533	10405738	< 2.2e-16
group II	23295652	1158	113	7517040	7507226	0.837
group III	16798506	1226	104	5325760	5764740	< 2.2e-16
group IV	32632948	1719	171	11075843	10983967	0.04622
group V	12251397	980	128	4198998	4022731	1.14e-13
group VI	17083675	965	93	5605389	5223090	< 2.2e-16
group VII	27937443	1726	183	9642342	10137264	< 2.2e-16
group VIII	19368704	1177	128	6508569	6477095	0.387
group IX	20249479	1374	149	6868532	6267635	< 2.2e-16
group X	15657440	1050	107	5286434	5883914	< 2.2e-16
group XI	16706052	1344	185	5543259	5455211	4.402e-05
group XII	18401067	1301	116	6049383	6006811	0.2558
group XIII	20083130	1303	137	6640756	6646041	0.8806
group XIV	15246461	984	94	5118033	5192484	0.06052
group XV	16198764	1026	114	5102727	5321175	2.422e-10
group XVI	18115788	1063	97	5635724	5357195	6.306e-12
group XVII	14603141	929	93	4897567	4834424	0.08998
group XVIII	16282716	1020	101	5251120	4896094	< 2.2e-16
group XIX	20240660	1373	132	6414729	6790731	< 2.2e-16
group XX	19732071	1259	113	5868160	5362585	< 2.2e-16
group XXI	11717487	599	71	3488145	2936194	< 2.2e-16

^*^The analysis was performed for each chromosome separately.

^**^The last column contains an indicator of the statistical significance of differences in the intergenic distance.

When comparing marine and freshwater specimens, statistically significant differences were found in the expression of 2,982 out of 22,456 annotated genes of G. *aculeatus* (significance level 95%). The expression of 1,304 genes was higher in marine stickleback, and the expression of 1,678 genes was higher in the freshwater form. *Table 2* shows 10 genes with the highest difference in expression in individuals of different ecotypes.

*Figure 4* graphically represents the results of the differential analysis of 50 genes whose level of expression is most significantly different in the three-spined stickleback experimental groups. It was shown (similarly to MDS-graph) that marine and freshwater specimens differ considerably in their level of expression of some genes (judging by the clustering of the samples at the top of the figure). Moreover, 50 of the analyzed genes are predominantly genes whose expression is enhanced in marine samples.

**Fig. 5:**
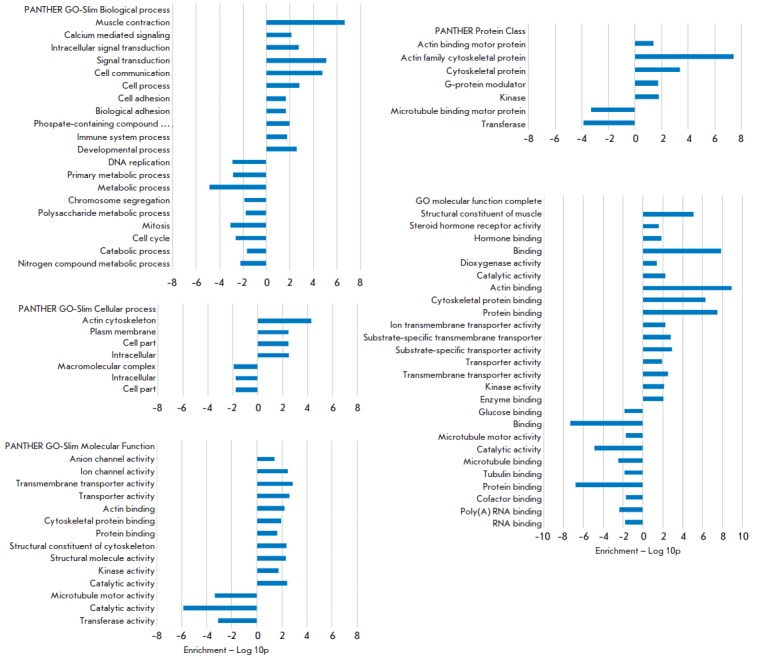
Statistically significant gene ontology terms for differently expressed genes in marine and freshwater stickleback gill samples. Increased expression level of specific functional categories in marine sticklebacks, in relation to freshwater sticklebacks, equals to the deviation of Enrichment -Log10p in positive value. The names of the database for GO categories are indicated in the upper left corner of each barplot

The results of the functional analysis are shown in *Fig. 5*. The genes which are UP-expressed (“overexpressed”) in the gills of the marine stickleback deviate to the right of the point of origin, while DOWN-expressed genes deviate to the left. UP- and DOWN-expressed genes can be interpreted as marine and freshwater ones, respectively. In addition, among the genes differentially expressed in the marine form, the content of genes associated with transmembrane functions and the cytoskeleton, e.g. those associated with the activity of ionic and anionic channels, transmembrane transporters, substrate-specific transmembrane transport activity, as well as other categories associated with membranes, proved significantly higher. This is quite logical and can be attributed to the fact that the maintenance of intracellular homeostasis in different osmotic conditions requires significant activity by transmembrane systems. Among the genes whose expression is increased in the freshwater form, there are many genes associated with the cell cycle: DNA replication, mitosis, chromosome segregation, as well as those associated with intracellular transport and microtubules. Differences in the processes of cell division can be associated with different rates of development of stickleback in the sea and in fresh water, which, in turn, can be defined by the temperature regime. However, this phenomenon requires further study and explanation.

The content of genes associated with muscle activity is increased among the differentially expressed genes of the marine form, which can be explained, for example, by the need for males of marine sticklebacks to migrate to the coast where the spawning takes place before the mating season, whereas in freshwater forms such movement is unnecessary, as spawning occurs directly in the habitat. The differences in the immune processes in the two forms of stickleback, apparently, may be due to differences in the freshwater and marine parasitic fauna that affects stickleback [28].

Our results only weakly correlate with the data for other fish species [9, 11-13, 15]. Is this related to the methodological features of the functional analysis of the gene lists or do different species adapt differently to saline conditions? This issue remains open and requires more in-depth studies. However, there is evidence in favor of the idea that the response to changes in osmotic conditions can be individual. For example, a study of changes in gene expression in the gills of two related arctic char larvae (*S. alpinus*) revealed 1,045 and 1,544 genes differentially expressed in each of these lines, respectively [16]. At the same time, only 257 genes were common; i.e. in less than a quarter of the genes responding to changes in osmotic conditions expression changed in a similar way. And this in representatives of just one species!

Based on the intergenic distances for a complete set of genes of three-spined stickleback and the distances between the genes participating in osmoregulation, the distribution of differentially expressed genes on the chromosome is indeed not accidental. For example, the distance between genes whose regulation varies with change in osmotic conditions does not statistically differ from the intergenic distances of other genes only in seven out of 21 chromosomes of three-spined stickleback (*Table 3*). This confirms the hypothesis that the genes in the eukaryotic genome are not distributed randomly but are combined into co-expressed clusters [17, 18]. This result suggests that we still do not know much about the structure of the eukaryotic genome.

The previously published results of the search for single nucleotide polymorphisms associated with marine and freshwater forms in the genome of threespined stickleback [3, 4] showed that such polymorphisms are predominantly localized in small parts of the genome called “divergence islands.” We compared the localization of the differentially expressed genes we had identified with the position of the “divergence islands” involved in the adaptation of stickleback to fresh water. Out of 2,982 differentially expressed genes, 28 were found in the islands of adaptive divergence, which is significantly higher than the number of random coincidences. All in all, there are 212 of the 29,245 annotated three-spined stickleback genes in the divergence islets (according to the Poisson test, at P-value is 0.0001). This fact seems quite natural, since if there are single nucleotide polymorphisms in certain loci that differ in the marine and freshwater specimens of three-spined stickleback, then it is logical to assume that the expression of genes in these loci will differ as well with rather high probability, because some polymorphisms can be in the regulatory elements of these genes.

## CONCLUSION

Summarizing the results presented in this work, let us emphasize that the use of modern methods of parallel sequencing to determine the activity of gene expression allowed us to identify an array of genes and the range of mechanisms involved in the process under study. Using the example of the adaptation of three-spined stickleback to changes in osmotic conditions, it has been shown that genes whose expression varies with the osmotic response are actively involved in such processes as regulation of the cell cycle, membrane transport, immunity, muscle contractions, etc. At the same time, a comparison of the enriched categories of differentially expressed genes with the results obtained earlier in other research centers reveals a low universality of the molecular mechanisms of adaptation to change in habitat conditions. This phenomenon requires further study.
